# ASO Author Reflections: Timing of Immediate Lymphatic Reconstruction—Expanding Access without Compromising Technical Success

**DOI:** 10.1245/s10434-026-20193-3

**Published:** 2026-07-15

**Authors:** Helen M. Johnson, Susie X. Sun, David M. Adelman, Edward I. Chang, Solange Cox, Shannon Foreman, Maggie Keener, Lauren Patterson, Simona F. Shaitelman, Kelly K. Hunt, Sarah M. DeSnyder

**Affiliations:** 1 Department of Breast Surgical Oncology, The University of Texas MD Anderson Cancer Center, Houston, TX USA; 2 Department of Plastic Surgery, The University of Texas MD Anderson Cancer Center, Houston, TX USA; 3 Department of Breast Radiation Oncology, The University of Texas MD Anderson Cancer Center, Houston, TX USA

## Past

Breast-cancer-related lymphedema (BCRL) is a dreaded long-term complication of breast cancer treatment. Immediate lymphatic reconstruction (ILR) has been demonstrated to decrease the risk of BCRL, with a 69% reduction in those with a mean of two reconstructed lymphatic channels.^1^ Despite demonstrated efficacy and increasing utilization of this risk-reducing procedure,^2^ barriers exist to widespread adoption of ILR, including lack of access to microvascular surgeons,^3^ high opportunity costs, and inconsistent insurance coverage.^4^

## Present

Our retrospective cohort study demonstrates that the technical success of ILR is not impacted by timing of axillary staging with sentinel lymph node dissection (SLND) or targeted axillary dissection (TAD), either concurrent with axillary lymph node dissection (ALND) or staged.^5^ A total of 866 patients undergoing ALND for whom ILR was planned had a > 90% technical success rate, with a median of 2 lymphatic channels reconstructed, regardless of prior SLND or TAD. These findings highlight opportunities to defer decision-making about completion ALND to multidisciplinary review of the final surgical pathology and potentially expand access to ILR.

## Future

Several challenges could be mitigated by staging the initial axillary procedure and ALND with ILR. One relevant challenge is lack of access to a microvascular surgeon who performs ILR. Given our findings, it would be reasonable for breast surgeons to perform axillary staging with a plan to refer patients out for completion ALND and ILR when indicated, thereby expanding access to this highly specialized procedure. For breast surgeons with access to a microvascular surgeon, SLND or TAD can be performed without use of intraoperative evaluation of the nodes with frozen section, with plans to schedule completion ALND and ILR at a later date, if indicated. This approach would optimize operating room efficiency by avoiding “standby” scheduling of the microvascular surgeon, ensuring that specialized equipment is only pulled when its use is definitively planned and facilitating more accurate estimation of case duration. As cancer survivorship expands, preventing long-term complications such as BCRL that impact quality of life is critical, and expanding access to preventative procedures should be a top priority.
